# An Overview of the Safety, Efficiency, and Signal Pathways of Stem Cell Therapy for Systemic Lupus Erythematosus

**DOI:** 10.1155/2021/2168595

**Published:** 2021-08-13

**Authors:** Qian Yang, Yiping Liu, Guangyong Chen, Wancong Zhang, Shijie Tang, Tianbiao Zhou

**Affiliations:** ^1^Department of Nephrology, Second Affiliated Hospital of Shantou University Medical College, Shantou 515041, China; ^2^Department of Plastic Surgery and Burn Center, The Second Affiliated Hospital of Shantou University Medical College, Shantou 515041, China; ^3^Plastic Surgery Institute of Shantou University Medical College, China

## Abstract

Systemic lupus erythematosus (SLE) is an autoimmune disease that affects multiple organs and tissues. Mesenchymal stem cells (MSCs) are considered a good source for autoimmune disease and hematological disease therapy. This review will summarize the efficacy, safety, and mechanisms of MSC therapy for SLE. MSC therapy can reduce anti-dsDNA, antinuclear antigen (ANA), proteinuria, and serum creatinine in SLE patients. In animal models of SLE, MSC therapy also indicates that it could reduce anti-dsDNA, ANA, proteinuria, and serum creatinine and ameliorate renal pathology. There are no serious adverse events, treatment-related mortality, or tumor-related events in SLE patients after stem cell treatment. MSCs can inhibit inflammatory factors, such as MCP-1 and HMGB-1, and inhibit inflammation-related signaling pathways, such as the NF-*κ*B, JAK/STAT, and Akt/GSK3*β* signaling pathways, to alleviate the lesions in SLE.

## 1. Introduction

Systemic lupus erythematosus (SLE) is an autoimmune disease characterized by overexpression of antibodies and activation of an inflammatory response that commonly affects the skin, joints, kidneys, and central nervous system and can lead to disability and death [1–3]. SLE is one of the top 20 leading causes of death in females between 5 and 64 years of age [3]. SLE is four times more prevalent in black women than in white women, and patients of African descent tend to develop disease earlier and have higher mortality [3]. Conventional therapy for SLE involves steroid and/or immunosuppressive drug treatment. However, conventional therapy is only effective in mild SLE patients, and the drugs have significant side effects over long-term use, such as infection, gastrointestinal syndrome, herpes zoster, or varicella [4–7]. Due to the high relapse and low survival rate of SLE, novel treatments have been investigated in recent years and some progress has been made.

Mesenchymal stem cell (MSC) transplantation is regarded as a novel treatment for SLE, and clinical research and animal experiments have been conducted with encouraging outcomes. MSCs can be derived from abundant sources, such as marrow, human umbilical cord, and adipose tissue [8]. MSCs have the potential to regulate the immune system and control inflammation. MSC treatment can alleviate the lesions of the glomerulus and renal interstitium and improve renal function with few adverse effects [9]. MSCs can modulate the inflammatory microenvironment and influence abnormal activation of signal pathways in SLE patients and lupus mice. They also modulate T lymphocyte proliferation and differentiation [10–14]. Thus, MSC therapy is a potential treatment for SLE patients. This review pays attention to the efficacy and safety of MSC therapy for SLE, based on both preclinical and clinical studies, and further discusses the immunomodulatory function which involves lymphocytes, cytokines, and signaling pathways.

### 1.1. The Efficacy of Mesenchymal Stem Cell Therapy for SLE: A Preclinical Model

The efficacy of MSC therapy in animal experiments was evaluated in recent years (Table 1). Liu et al. [15] found that human placenta-derived MSC treatment could improve the renal function and decrease the proteinuria levels of MRL/lpr mice. In addition, anti-double-stranded DNA (anti-dsDNA) antibody levels decreased in the MSC group compared to a control group treated with normal saline. The immune complex immunoglobulin G (IgG) deposited in glomeruli was observed to be significantly less in the MSC group when compared with the control group. Thus, the renal destruction was alleviated and the glomerular swelling and lymphocyte infiltration were slightly less than before MSC treatment. The deposition of electron dense material in the glomeruli was also reduced. Ruan et al. [16] gave weekly intravenous (IV) injections of human umbilical cord mesenchymal stem cells (H-UC-MSCs) for 4 weeks to B6.Fas mice and showed that the levels of anti-nuclear, anti-histone, and anti-dsDNA antibodies in the MSC treatment group decreased compared with the B6.Fas mouse model group. Renal function improved as the 24-hour urine protein was lower in the MSC treatment group than the lupus control group. As for renal pathology, the proliferation of glomerular mesangial cells and tubulointerstitial fibrosis were reduced following MSC treatment, as well as lowering immune complex deposition in the glomeruli compared to the lupus group. MSC transplantation ameliorated mouse lupus by decreasing the 24 h proteinuria and serum anti-dsDNA antibody [11, 16, 17]. Ma et al. discovered that H-UC-MSC treatment improved renal function, decreased proteinuria and plasma levels of Cr by inhibiting C5 activation, which is involved in the complement system, and can induce an inflammatory response in lupus nephritis [18]. Researchers have shown that MSC therapy diminishes the deposition of immune complexes and complement components (IgG, IgA, and C3) in lupus glomeruli [18]. As a result, inflammatory infiltration and mesangial hyperplasia of the glomeruli decrease after MSC treatment in lupus mice [10, 18]. Zhang et al. [19] found decreased lymphocyte infiltration, mesangial cell proliferation, and interstitial fibrosis in glomeruli following treatment with adipose-derived mesenchymal stem cells (ADMSC). ADMSC treatment could also decrease the proteinuria, serum anti-dsDNA antibody, and serum Cr. Zhang et al. showed that after MSC treatment, the level of the anti-dsDNA antibody exhibited a declining trend compared with other groups [20]. Mononuclear cell infiltration and the deposition of IgG in glomeruli and tubules also decreased. Sun et al. infused allogenic bone marrow mesenchymal stem cells (BMMSCs) into MRL/lpr mice at the early stage and the mature stage [21]. The serum level of the anti-dsDNA antibody, immunoglobulin, serum albumin, and ANA was reduced at both these stages. In addition, they found that C3 and IgG deposition decreased in the kidney after MSC treatment. Similar to the above studies, Ji et al. showed that BMMSC transplantation can decrease the levels of anti-dsDNA, IgG, IgM, ANA, and proteinuria [22]. However, they showed that at high dose, MSCs had the effect on immunosuppression and production of proteinuria, whereas the low dose had no effect. Jang et al. [13] found that infusion of BMMSCs during the preclinical phase could delay the time when the anti-dsDNA antibody increased and lupus nephritis progressed. As for the mice that had already developed nephritis, infusion of MSCs could reduce the level of the anti-dsDNA antibody, the deposition of immune complexes and complement factors (C3), and the infiltration of CD4+ T cells, as well as B220+ B cells, in the kidneys [13]. Zhou et al. found that the levels of anti-dsDNA antibodies, serum Cr, and 24 h proteinuria decreased following MSC treatment [23]. BMMSCs ameliorated late-stage renal pathology by reducing the expression of vascular endothelial growth factor (VEGF), transforming growth factor-*β* (TGF-*β*), and fibronectin (FN) in the kidney [23]. Thus, MSCs can regulate cytokines to delay the development of renal fibrosis and decrease the level of proteinuria.

### 1.2. Clinical Efficacy and Safety of Stem Cell Therapy for SLE

#### 1.2.1. Clinical Efficacy of Stem Cell Therapy for SLE

MSCs have been tentatively applied for SLE treatment in recent years. Various clinical research studies have shown MSC therapy to have encouraging potential for this specific immune disease. Barbado et al. intravenously injected three SLE patients with MSCs (1.5 million MSCs/kg) and found that the level of proteinuria decreased below 0.6 g/24 h at 1 month after MSC treatment when compared with baseline [24]. Serum creatinine (Cr) levels of two of the patients also decreased while the serum Cr of the third patient was normal throughout the entire study. Leng et al. assessed the efficacy of MSC treatment in a 10-year follow-up study that enrolled 24 Chinese patients with severe SLE [25]. All patients were treated with the combination of high-dose immunosuppressive therapy with autologous peripheral blood stem cell transplantation. More than half of the patients completed the 10-year follow-up, and their median proteinuria level decreased from 4.00 g/24 h before treatment to 0.00 g/24 h at 5 years after transplantation, which was maintained until the end of the 10-year follow-up. Liang et al. evaluated serum indices and renal function of 15 refractory SLE patients with MSC treatment (bone marrow mesenchymal stem cells (BMMSCs)) [26]. The follow-up study demonstrated that sera from 11 of 15 patients had anti-dsDNA antibody levels that decreased below baseline at both 1 and 3 months after transplantation. The 24 h proteinuria of nearly all patients except one decreased significantly at 1 week from 2538.0 ± 382.3 to 1430.7 ± 306.3 mg and further decreased continually at the 1-, 3-, 6-, and 12-month follow-ups. Another short-term clinical study also showed recovery of renal function of SLE patients with both 24 h urine protein and serum Cr decreasing at the 12- to 18-month follow-up [21]. One case reported a refractory SLE patient suffering from several complications, such as arthritis, skin vasculitis, and high titers of anti-dsDNA antibody, who was treated with high-dose immune suppression medication and stem cell transplantation. The serum Cr and proteinuria were decreased at follow-up 9 months posttransplantation, and the anti-dsDNA titer dropped to undetectable levels [27]. Some other case reports also revealed that refractory SLE patients who had been treated with conventional immunosuppressants and glucocorticoids with no response, and their condition steadily declined. After programing stem cell transplantation therapy, their anti-dsDNA and serum anti-nuclear antibodies decreased [28–31]. The results also indicated that Cr and Cr clearance became normal as well [32].

#### 1.2.2. The Safety of Stem Cell Therapy in SLE

The safety of stem cell therapy is important and is an essential precondition for clinical application. The safety of MSC clinical applications is still the most concerning issue. In the past years, MSCs were used for the treatment of liver diseases [33], diabetes mellitus [34], and idiopathic Parkinson's disease [35], with no significant adverse effects reported in most clinical trials. Stem cell therapy for SLE has less adverse effects compared with conventional treatment (steroid and/or and immunosuppressive drugs). According to the data from some follow-up studies and case reports, there were no serious adverse events [21, 36–38], treatment-related mortality [36], or tumor-related events [36] after stem cell treatment of SLE patients [39]. In a long-term follow-up study that enrolled 81 refractory SLE patients, 11 infection events occurred in patients with MSC treatment [40]. The immediate adverse events after MSC infusion were mild and included dizziness and a warm sensation that disappeared in a short time. Delayed adverse events were mainly bacterial and viral infections during follow-up and most resolved. However, as advanced and refractory SLE patients suffered from multiple system dysfunction, it is doubtful that the delayed infection events were caused by MSC treatment [37]. An observational study demonstrated that mortality of severe SLE patients after transplantation mainly included original disease, transplant-related complications, and infections [41]. The transplant-related mortality was mainly caused by the transplant centers' lack of experience and the severity of disease [41]. As for child-bearing SLE patients, MSC therapy reduced the activity of lupus and risk of pregnancy complications. MSC therapy also improved the fetal outcome when compared with traditional treatment [42].

### 1.3. Effect of MSCs on Inflammatory Factors and Potential Signaling Pathways of MSCs in SLE

MSC therapy was considered an appropriate treatment for autoimmune diseases like SLE, because MSCs exhibit immunosuppressive potential only in an inflammatory environment [43]. MSCs promote anti-inflammatory actions and inhibit proinflammatory progression in SLE and regulate the imbalance of the inflammatory response [44]. Monocyte chemoattractant protein-1 (MCP-1) and high-mobility group box chromosomal protein 1 (HMGB-1) are proinflammatory cytokines that are correlated with renal pathogenesis in SLE [45]. MSCs can reduce the overexpression of MCP-1 and HMGB-1 mRNA and decrease the serum and urine level of these two proinflammatory cytokines to delay the kidney damage [45].

Furthermore, Choi et al. [46] found that MSC transplantation increased serum levels of interleukin-4 (IL-4) and interleukin-10 (IL-10) when compared with the control group. The study revealed that the anti-inflammatory action and the regulation of the T cell subset ratio by MSCs relied on hypoxia-inducible factor 1*α* (HIF-1*α*) and mammalian target of rapamycin (mTOR) [43]. mTOR and HIF-1*α* play a critical role in regulating cell growth and proliferation (e.g., Th17 cells), which are related to the abnormal development of T cells in lupus mice and SLE patients [43]. The Th17 cells promote an inflammatory response by secreting interleukin-17 (IL-17) [12, 47]. MSCs modulate the proportion of Th17 cells by cell-cell contact [47]. IL-17 is a proinflammatory agent that infiltrates glomeruli and causes the destruction of renal tissue in part [43]. IL-17 can also promote various cells to secrete inflammatory cytokines [12]. It has been suggested that ADMSC treatment inhibits the mTOR pathway and the expression of HIF-1*α*, as well as the expansion of Th17 cells. Thus, the secretion of IL-17 by Th17 cells is decreased in the lupus kidney, which reduces the progression of lupus [43]. Moreover, MSCs also downregulate Th17 cells and IL-17 to suppress the inflammatory response by secreting several cytokines, including IL-10, prostaglandin E2 (PGE2), and TGF-*β* [12]. TFN-*γ*, which exists in the internal environment of lupus mice, upregulates the secretion of these cytokines. These cytokines play an important role in reducing Th17 cell differentiation and proliferation directly or indirectly [12]. In contrast, interleukin-23 (IL-23) plays a crucial role in Th17 cell differentiation and function [47]. ADMSC downregulates the expression of IL-23 mRNA level, thereby reducing the level of IL-23 [47]. MSC therapy promotes the apoptosis of abnormal T cells in lupus and regulates the number of Th1 and Th2 cells [11]. Imbalance of Th1 and Th2 cells has been observed in SLE patients and mouse models of lupus [44]. Th1 cells are proinflammatory cells that produce interferon-*γ* (IFN-*γ*) and IL-2, whereas Th2 cells are anti-inflammatory cells that produce IL-4, IL-5, IL-6, and IL-10 [23, 44]. MSCs decrease Th1 proinflammatory cytokines and increase Th2 anti-inflammatory cytokines [44]. They also reduce other inflammatory cytokines such as tumor necrosis factor-*α* (TNF-*α*) and IL-12, which play a role in the proinflammatory response and inhibition of Th2 cells [44]. T follicular helper (Tfh) cells are a subtype of CD4+ T helper cells that contribute to the pathogenesis and symptoms of SLE [20]. The increased proportion of Tfh cells was observed in lupus mice and SLE patients [13, 20]. The overactivation of Tfh cells in SLE results in the overexpression of C-X-C chemokine receptor 4, programmed cell death-1, and interleukin-21. These molecules influence the differentiation of B cells [20]. Studies show that MSCs downregulate the percentage of Tfh cells [19] through inhibiting the Tfh cell proliferation and differentiation of naïve T cells [20] and decreasing their circulating precursors [13]. The inhibition of Tfh cells is mediated by iNOS. The activation of iNOS involves the nuclear factor kappa-B (NF-*κ*B), STAT, and Akt signaling pathways via direct cell-to-cell contract of MSCs and CD4+ T cells [20]. Tfh cells have the capacity to develop long-lived plasma cells. Long-lived plasma cells exist in an inflammatory environment and cause prolonged destruction of organs, which makes SLE symptoms difficult to ease. Thus, the reduction of Tfh cells can lead to a reduction in long-lived plasma cells [13].

**Figure 1:**
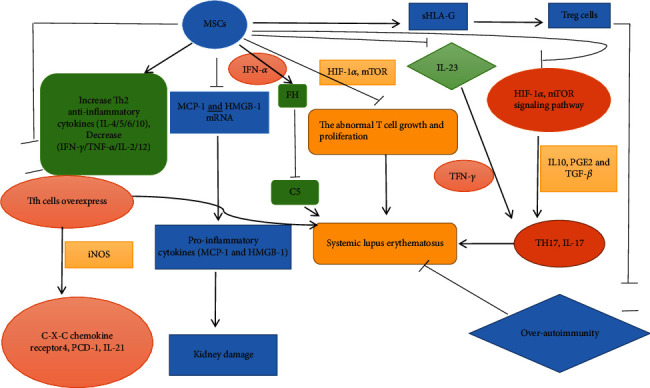
Effect of MSCs on cytokine production in systemic lupus erythematosus.

The abnormal activation of the complement system and deposition of immune complexes also promote the progression of lupus nephritis. C3 and C5 are important molecules of the complement system [18]. Ma et al. demonstrated that MSCs inhibit the activation of C5 by secreting factor H (FH) [18]. The IFN-*α*, produced by SLE cells, promotes FH secretion by MSCs. FH suppresses the activation of C5 by the cleavage of C3 and competitive combination with activators of the classical pathway. Tolerogenic regulatory T cells (Tregs), a special subtype of CD4+ T cells, which can suppress immune responses and proinflammatory cytokine production, are reduced in SLE. Many studies have demonstrated that MSCs can upregulate Treg cells to modulate excessive autoimmunity [16, 21, 46, 48, 49]. Furthermore, MSCs increase the Treg cells of SLE patients via secreting soluble human leukocyte antigen-G (sHLA-G), a nonclassical HLA class I molecule that is involved in immunosuppression (Figure 1) [48].

Stem cell therapy for SLE is associated with signaling pathways such as the NF-*κ*B [15], STAT [39], and Akt/GSK3*β* signaling pathways [22], which are related to the synthesis of downstream inflammatory mediators [15] and the regulation of T cells, including Th1, Th2, Treg, and Tfh cells [20, 22, 50].

NF-*κ*B is a transcription factor for many inflammatory cytokines. NF-*κ*B can efficiently induce the expression of inflammatory cytokines (IL-1, IL-6, and TNF-*α*), adhesion molecules (vascular cell adhesion molecule-1 (VCAM-1); intercellular cell adhesion molecule-1 (ICAM-1)), chemokines, and inflammatory enzymes (iNOS, COX-2). It has been demonstrated that the inflammatory microenvironment of SLE patients with lupus nephritis increases NF-*κ*B expression in the glomerular endothelial and mesangial cells, and overexpression of NF-*κ*B could induce expression of inflammatory cytokines, chemokines, adhesion molecules, and inflammatory enzymes in turn, causing a vicious cycle [15]. MRL/lpr mice were treated with human placenta-derived mesenchymal stem cells (pMSCs) to reduce NF-*κ*B mRNA and the protein level of phospho-NF-*κ*B p65 and their protein synthesis and to further downregulate NF-*κ*B signaling pathway activation. Thus, the expression of downstream TNF-*α*, plasminogen activator inhibitor-1 (PAI-1), and ICAM-1 decreases in MRL/lpr mouse kidneys [15]. PAI-1 is a proinflammatory cytokine that is related to a hypercoagulable state of blood and causes glomerular microthrombi. Therefore, MSC treatment could suppress abnormal NF-*κ*B signal pathway activation to reduce the inflammatory microenvironment and ameliorate lupus symptoms.

CD1c+ DCs are a subset of dendritic cells that can suppress the proliferation and differentiation of T cells and regulate T regulatory cells and Th2 cells in an IL-10-dependent manner in various organs such as the liver. Research shows that specific CD1c+ DCs might play an important role in ameliorating immune dysfunction of SLE patients [39]. The number of CD1c+ DCs is negatively correlated with the activity and severity. UC-MSC transplantation therapy can upregulate CD1c+ DC numbers through FLT3L (Fms-related tyrosine kinase 3-ligand), a regulator that binds to FLT3 to stimulate DC proliferation. Lupus CD8+ T cells produce IFN-*γ*, and this cytokine enhances the expression of FLT3L, in MSCs, in a manner that is mediated by the JAK/STAT signaling pathway. AG490 inhibits the JAK/STAT signaling pathway, causing the number of CD1c+ DCs as well as the expression of FLT3L to decrease [39].

STAT3 is a protein that is encoded by a gene on chromosome 17 and contributes to Tfh cell differentiation. It is demonstrated that BMMSCs can inhibit the gene expression and phosphorylation of STAT3 to prevent naïve T cells from differentiating to Tfh cells [50]. Recent research has also found that abnormal proliferation and activation of autoreactive T cells play an important role in the development of SLE [20, 22, 43].

**Figure 2:**
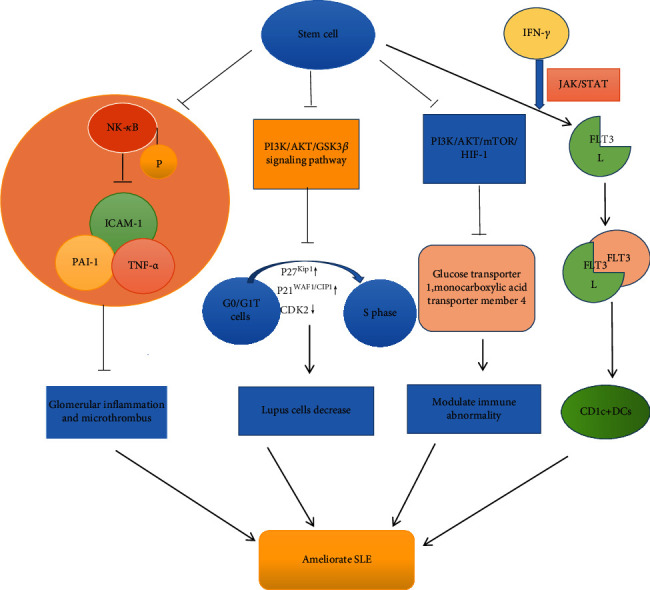
Stem cell therapy influences abnormal activation of signal pathways in systemic lupus erythematosus.

High-dose MSC transplantation can suppress the activation of lupus T cells through the Akt/GSK3*β* signaling pathway to further modulate the immune disorder and ameliorate SLE abnormalities [22]. PI3K is the major upstream molecule that activates Akt to influence diverse downstream targets such as GSK3*β*, mTOR, and p2, which can regulate the cell cycle [22]. In MRL/lpr mice, activation of the PI3K/Akt/GSK3*β* signaling axis is prevented and lupus T cells accumulate in G0/G1 while the number of S phase T cells decreases. This suggests that MSC treatment influences lupus T cells by increasing the activity of p27^Kip1^ and p21^WAF1/CIP1^ and decreasing the activity of CDK2, which is a cyclin-dependent kinase associated with the cell cycle progression through G1. This study reveals that high-dose MSC treatment has a negative effect on cell growth of lupus T lymphocytes and could inhibit G1/S transition of abnormal T cells via inhibition of the PI3K/Akt/GSK3*β* signaling pathway in the abnormal T cells [22]. The mTOR is a downstream target of Akt, and it increases the HIF-1*α* protein level by promoting HIF-1*α* mRNA transcription. HIF-1*α* plays an important role in Th17 cell differentiation and IL-17 expression. It acts on its target cytokines as well as monocarboxylic acid transporter member 4 and glucose transporter 1 and various glycolytic enzymes to regulate aerobic glycolysis in Th17 cells. The suppression of Akt indirectly decreases the expression of mTOR and HIF-1*α*, thereby reducing Th17 cells and IL-17 in glomeruli and tubules, supporting the model that MSC treatment suppresses the activation of the Akt/mTOR/HIF-1*α*/Th17 pathway to modulate immune abnormality in SLE (Figure 2) [43].

## 2. Conclusions

Stem cell transplantation treatment has been studied as an alternative therapy for autoimmune disease in recent years. Traditional therapy has little effect on refractory SLE patients and has accumulating drug toxicity, as well as causing drug complications. Stem cell therapy decreases the level of serum autoantibodies (anti-dsDNA, ANA), proteinuria, and Cr and improves lupus renal pathology and reduces the inflammatory response in SLE patients, as well as animal models. There are no serious adverse events, treatment-related mortality, or tumor-related events in SLE patients after stem cell treatment. MSCs can inhibit inflammatory factors, such as MCP-1 and HMGB-1, and inhibit activation of the NF-*κ*B, JAK/STAT, and Akt/GSK3*β* signaling pathways to alleviate the lesions in SLE. In addition to the studies above, the different effects of different types of stem cells need to be studied in the future.

## Figures and Tables

**Table 1:** Efficacy of MSC therapy in animal experiments.

Author, year	Stem cell type	Animal model	Groups	Handing methods	Treatment effect
Liu et al. [15]	pMSCs	MRL/lpr mice, BALB/C mice	The control group (*n* = 10) The vehicle group (*n* = 10) The LEF group (*n* = 10) The MSC group (*n* = 10)	(pMSCs) 1 × 10^6^ cells via the tail vein	Proteinuria ↓, anti-dsDNA ↓, IgG deposition ↓, renal injury ↓
Ruan et al. [16]	H-UC-MSCs	B6.Fas mice	Normal control (C57BL/6 mice) group (*n* = 10), model (B6.Fas mice) group (*n* = 10), low-dose treatment (B6.Fas mice) group (*n* = 10), medium-dose treatment (B6.Fas mice) group (*n* = 10), high-dose treatment (B6.Fas mice) groups (*n* = 10)	High: 2 × 10^6^ cells via the tail vein Medium: 1 × 10^6^ cells via the tail vein Low: 0.5 × 10^6^	Anti-nuclear ↓, anti-histone ↓, anti-dsDNA antibody ↓, proliferative mesangial cells ↓, tubulointerstitial fibrosis ↓, 24 h proteinuria ↓
Ma et al. [18]	UC-MSCs	B6.lpr mice	MSC group (*n* = 8) C5aRA, Merck group (*n* = 8) Control group (*n* = 8)	MSCs (1 × 10^6^) via the tail vein	Proteinuria ↓, plasma levels of creatinine ↓, mesangial matrix ↓, inflammatory cell infiltration ↓, IgG ↓, IgA ↓, C3 ↓
Zhang et al. [19]	ADSCs	MRL/lpr mice	Control group (*n* = 5) ADSC group (*n* = 5) CTX group (*n* = 5)	ADSCs (1 × 10^6^) via the tail vein	Proteinuria ↓, serum anti-dsDNA antibody ↓, serum creatinine ↓, renal pathology ↓
Zhang et al. [20]	H-UC-MSCs	B6.lpr mice	MSC group FLS group Phosphate-buffered saline (PBS) treatment group	UC-MSCs (1 × 10^6^) via the tail vein	Anti-dsDNA antibody ↓, IgG ↓, mononuclear cell infiltration ↓
Sun et al. [21]	BMMSCs	MRL/lpr mice	BMMSC group (*n* = 12) Control group (*n* = 12) CTX group (*n* = 12)	BMMSCs (0.1 × 10^6^ cells per 10 g body weight) via the tail vein	Anti-dsDNA antibody ↓, immune globulin ↓, serum albumin ↓, ANA ↓, C3 ↓, IgG ↓
Ji et al. [22]	BMMSCs	MRL/lpr mice	Low-dose MSC group (*n* = 10) High-dose MSC group (*n* = 10) PBS group (*n* = 10) Control group (*n* = 10)	BMMSCs (0.05 × 10^6^ cells per 10 g body) MSC (0.2 × 10^6^ cells per 10 g body)	Anti-dsDNA antibody ↓, IgG ↓, IgM ↓, ANA ↓, proteinuria ↓
Jang et al. [13]	BMMSCs	NZB/W mice	BMMSC group (*n* = 10-11) PBS group (*n* = 10-11)	BMMSCs (1 × 10^6^) via retro-orbital injection of the venous sinus	Anti-dsDNA antibody ↓, immune complexes and complement factors ↓, CD4+ T cells ↓, B220+B cells ↓
Zhou et al. [23]	BMMSCs	MRL/lpr mice	Control group (*n* = 5) CTX-treated group (*n* = 4) MSC group (*n* = 4) MSC+CTX group (*n* = 4)	BMMSCs (1 × 10^6^) via the tail vein	Anti-dsDNA antibody ↓, serum creatinine ↓, 24 h proteinuria ↓

## Data Availability

Data sharing is not applicable to this article, as no datasets were generated or analyzed during the current study.
